# Spatial navigation is associated with subcortical alterations and progression risk in subjective cognitive decline

**DOI:** 10.1186/s13195-023-01233-6

**Published:** 2023-04-25

**Authors:** Qian Chen, Futao Chen, Cong Long, Yajing Zhu, Yaoxian Jiang, Zhengyang Zhu, Jiaming Lu, Xin Zhang, Zuzana Nedelska, Jakub Hort, Bing Zhang

**Affiliations:** 1 Department of Radiology, Drum Tower Hospital, Clinical College of Nanjing Medical University, Nanjing, 210008 China; 2 Institute of Medical Imaging and Artificial Intelligence, Nanjing University, Nanjing, China; 3 Medical Imaging Center, Affiliated Drum Tower Hospital, Medical School of Nanjing University, Nanjing, China; 4 Department of Radiology, The Affiliated Drum Tower Hospital of Nanjing University Medical School, Nanjing, China; 5 Memory Clinic, Department of Neurology, 2nd Faculty of Medicine, Charles University, University Hospital Motol, Prague, Czechia; 6 Jiangsu Key Laboratory of Molecular Medicine, Nanjing, China; 7 Institute of Brain Science, Nanjing University, Nanjing, China

**Keywords:** Spatial navigation, Subjective cognitive decline, Basal forebrain, Structural covariance network, Progression risk

## Abstract

**Background:**

Subjective cognitive decline (SCD) may serve as a symptomatic indicator for preclinical Alzheimer’s disease; however, SCD is a heterogeneous entity regarding clinical progression. We aimed to investigate whether spatial navigation could reveal subcortical structural alterations and the risk of progression to objective cognitive impairment in SCD individuals.

**Methods:**

One hundred and eighty participants were enrolled: those with SCD (*n* = 80), normal controls (NCs, *n* = 77), and mild cognitive impairment (MCI, *n* = 23). SCD participants were further divided into the SCD-good (G-SCD, *n* = 40) group and the SCD-bad (B-SCD, *n* = 40) group according to their spatial navigation performance. Volumes of subcortical structures were calculated and compared among the four groups, including basal forebrain, thalamus, caudate, putamen, pallidum, hippocampus, amygdala, and accumbens. Topological properties of the subcortical structural covariance network were also calculated. With an interval of 1.5 years ± 12 months of follow-up, the progression rate to MCI was compared between the G-SCD and B-SCD groups.

**Results:**

Volumes of the basal forebrain, the right hippocampus, and their respective subfields differed significantly among the four groups (*p* < 0.05, false discovery rate corrected). The B-SCD group showed lower volumes in the basal forebrain than the G-SCD group, especially in the Ch4p and Ch4a-i subfields. Furthermore, the structural covariance network of the basal forebrain and right hippocampal subfields showed that the B-SCD group had a larger Lambda than the G-SCD group, which suggested weakened network integration in the B-SCD group. At follow-up, the B-SCD group had a significantly higher conversion rate to MCI than the G-SCD group.

**Conclusion:**

Compared to SCD participants with good spatial navigation performance, SCD participants with bad performance showed lower volumes in the basal forebrain, a reorganized structural covariance network of subcortical nuclei, and an increased risk of progression to MCI. Our findings indicated that spatial navigation may have great potential to identify SCD subjects at higher risk of clinical progression, which may contribute to making more precise clinical decisions for SCD individuals who seek medical help.

**Supplementary Information:**

The online version contains supplementary material available at 10.1186/s13195-023-01233-6.

## Introduction

Alzheimer’s disease (AD) is a major global concern [1]. Subjective cognitive decline (SCD) is defined as self-experienced worsening of cognitive function without objectively detected deficits [2, 3]. Abundant evidence has shown that SCD may serve as a symptomatic indicator for preclinical AD in terms of amyloid pathology, cortical thinning, abnormal functional connectivity, and white matter degeneration [4–6]. However, SCD is a heterogeneous entity in terms of underlying etiology and clinical progression [7]. For example, memory complaints could be related to psychiatric disorders (e.g., depression, anxiety, and sleep disturbances), personality traits (e.g., neuroticism), metabolic diseases (e.g., diabetes), or neurodegenerative diseases (e.g., AD). In addition, the trajectories of objective cognitive function in SCD also show different patterns over time. Some SCD occurs with objective cognition remaining stable, while some SCD occurs with objective cognition gradually deteriorating into dementia [7]. Recent efforts have aimed at identifying those with SCD who are at higher risk of clinical progression to objective cognitive impairment [8–11]. This may contribute to making more precise clinical decisions for SCD individuals who seek medical help [7].

Spatial navigation is an essential ability for people to determine and maintain a route from one place to another using their own position and environmental clues [12]. Previous studies have shown spatial navigation impairment in AD dementia patients, which may be attributed to the widespread neurodegeneration involving the medial temporal, frontal, and parietal lobes. Spatial navigation impairment is also commonly observed in patients with mild cognitive impairment (MCI) typically due to AD [13–15]. However, the integrity of spatial navigation in the preclinical AD stage has not been fully investigated and is considered an overlooked cognitive marker [16]. Spatial navigation complaints are more frequently observed in SCD subjects than the normal controls (NCs) [17]; in accordance with this, our previous study has shown that the SCD group was outperformed by the NC group in the spatial navigation test [18]. These cross-sectional studies have provided preliminary evidence of the potential of spatial navigation in identifying incipient AD patients. Furthermore, longitudinal studies have suggested the promising utility of baseline spatial navigation in predicting future cognitive decline [19, 20]. Spatial navigation showed better performance than episodic memory in discriminating progressors from nonprogressors measured by the Clinical Dementia Rating scale [19]. Considering the heterogeneity of the SCD entity, we speculated whether the baseline spatial navigation performance could also serve as a predictor for future clinical progression in SCD subjects.

Cortical and subcortical atrophy and deformation have been commonly observed in MCI and AD patients and are recognized as a well-established imaging marker for the AD continuum [21–23]. The imaging findings of cortical morphometry in the SCD stage remain controversial; however, studies have converged to suggest that SCD is associated with subcortical volume reductions in the Ch4p subregion of the basal forebrain and the CA1 subregion of the hippocampus [4, 24, 25]. As suggested by histological studies, subcortical nuclei were affected by tau-related pathology since the earliest stages of AD [26]. Furthermore, subcortical nuclei such as the basal forebrain, hippocampus, caudate, and thalamus play important roles in cognition and spatial navigation [27, 28]. Significant associations between volumetric and functional measures of subcortical nuclei and spatial navigation have been observed [13, 29, 30]. Therefore, investigations of subcortical alterations may increase our understanding of the neural basis underlying cognitive and behavioral deficits in the SCD stage.

Moreover, graph theory analyses have provided a new perspective for investigating the neural mechanisms underlying neurological disorders and behavioral impairments [31, 32]. The gray matter network derived from the high-resolution T_1_-weighted (T_1_W) images can be constructed by calculating the structural covariance between pairs of regions [33, 34]. A characteristic small-world loss in the network constructed by cortical thickness and sulcal depth has been observed in MCI patients [35]. Alterations in structural covariance of hippocampal subregions and weakened transmission efficiency have been shown in AD patients [36]. Previous studies using the graph theory approach in SCD individuals were mainly focused on functional imaging and diffusion tensor imaging data [6, 37, 38]. Studies based on structural images have indicated a reorganized structural covariance network constructed by the regions in the Automated Anatomical Labelling (AAL) atlas in SCD subjects, which was associated with a steeper cognitive decline and an increased risk of clinical progression [39–41]. However, the topological properties of the structural covariance network of subcortical structures in SCD subjects, and the associations with spatial navigation, remain poorly understood.

In the present study, we aimed to investigate whether spatial navigation could reveal subcortical structural alterations and the risk of progression to MCI in SCD subjects. The SCD subjects were divided into two subgroups based on their spatial navigation performance. We expected to demonstrate the differences in the baseline volumes of subcortical nuclei and topological properties of the subcortical structural covariance network between the two groups. More importantly, we hypothesized that the SCD group with bad spatial navigation performance would be more likely to convert to MCI than the SCD group with good spatial navigation performance.

## Methods

### Participants

A total of 180 right-handed participants were enrolled in the present study. Of these, 77 were NCs, 80 were SCD subjects, and 23 were MCI patients. The inclusion criteria were 55–80 years old and having 8 or more years of education. Participants with a history of stroke, other neurological disorders that could lead to cognitive impairment (Parkinson’s disease, encephalitis, epilepsy, brain tumors, etc.), severe anxiety or depression, and contraindications for magnetic resonance imaging (MRI) were not enrolled. Subjects who complained of memory decline within the last 5 years and expressed worries associated with memory decline and did not meet the diagnostic criteria for MCI were defined as SCD [2]. Subjects with no cognitive complaints and associated worries and did not meet the diagnostic criteria for MCI were recruited as NCs. MCI patients were diagnosed by the criteria proposed by Jak et al. [42]. The detailed diagnostic criteria could be referred to the protocol for the Sino Longitudinal Study on Cognitive Decline (SILCODE) [43].

The study was conducted in accordance with the Declaration of Helsinki and was approved by the Ethics Committee of Nanjing Drum Tower Hospital. All participants signed an informed consent statement after gaining a sufficient understanding of the study procedure.

### Neuropsychological evaluation

All participants underwent a set of standardized neuropsychological evaluations, including SCD-questionnaire (SCD-Q) [43] and mini-mental state examination (MMSE) [44]. Memory function was assessed by the auditory verbal learning test (AVLT), including the immediate, short-delayed, long-delayed, cued recall, and recognition memory [45]. Executive function was assessed by the trail making test part A (TMT-A), trail making test part B (TMT-B) [46], symbol digit modalities test (SDMT) [47], and clock drawing test (CDT) [48]. Language function was assessed by the animal fluency test (AFT) [49] and the Boston naming test (BNT) [50]. The averaged *Z*-score of the cognitive tests was calculated as the composite score of the corresponding cognitive domain.

### Spatial navigation assessment

Spatial navigation ability was measured by the Amunet test battery (NeuroScios, Austria, Gmbh), which used a similar testing paradigm as the hidden goal task [51]. The test battery has been proven to be highly consistent with real space navigation [13, 52]. The description of the Amunet test battery and the schematic of the paradigm is detailed in our previous study [18]. Briefly, both egocentric and allocentric navigation strategies were assessed. In egocentric navigation, the examinees could only locate the hidden goal using their starting position towards the goal (the first-person perspective); however, in allocentric navigation, the examinees could only locate the hidden goal by its relationship with the external orienting cues but not the starting position. The egocentric and allocentric navigation subtasks both consist of 8 trails, and the average distance errors (from the position located by the examinee to the correct position of the goal) across all trails were recorded automatically. Notably, a lower distance error indicated a better navigation performance, and the test was no time limit. According to the average distance errors across all the egocentric and allocentric navigation trails, the SCD subjects were further symmetrically divided into the SCD-good (G-SCD) group (*n* = 40) and the SCD-bad (B-SCD) group (*n* = 40). The former group performed better than the latter group.

### Imaging data acquisition

The T_1_W images were obtained using two Philips 3 T MRI scanners. Participants wore earplugs and foam pads to abate noise and prevent head motion. The images in the Achieva TX were acquired with repetition time (TR) = 9.74 ms, echo time (TE) = 4.60 ms, and 192 sagittal slices, and those in the Ingenia CX were acquired with TR = 8.10 ms, TE = 3.70 ms, and 196 sagittal slices. The two scanners share the following parameters: slice thickness = 1 mm, field of view (FOV) = 256 × 256 mm^2^, and voxel size = 1 × 1 × 1 mm^3^.

### Volume extraction of the subcortical nuclei and hippocampal subfields

**Fig. 1:**
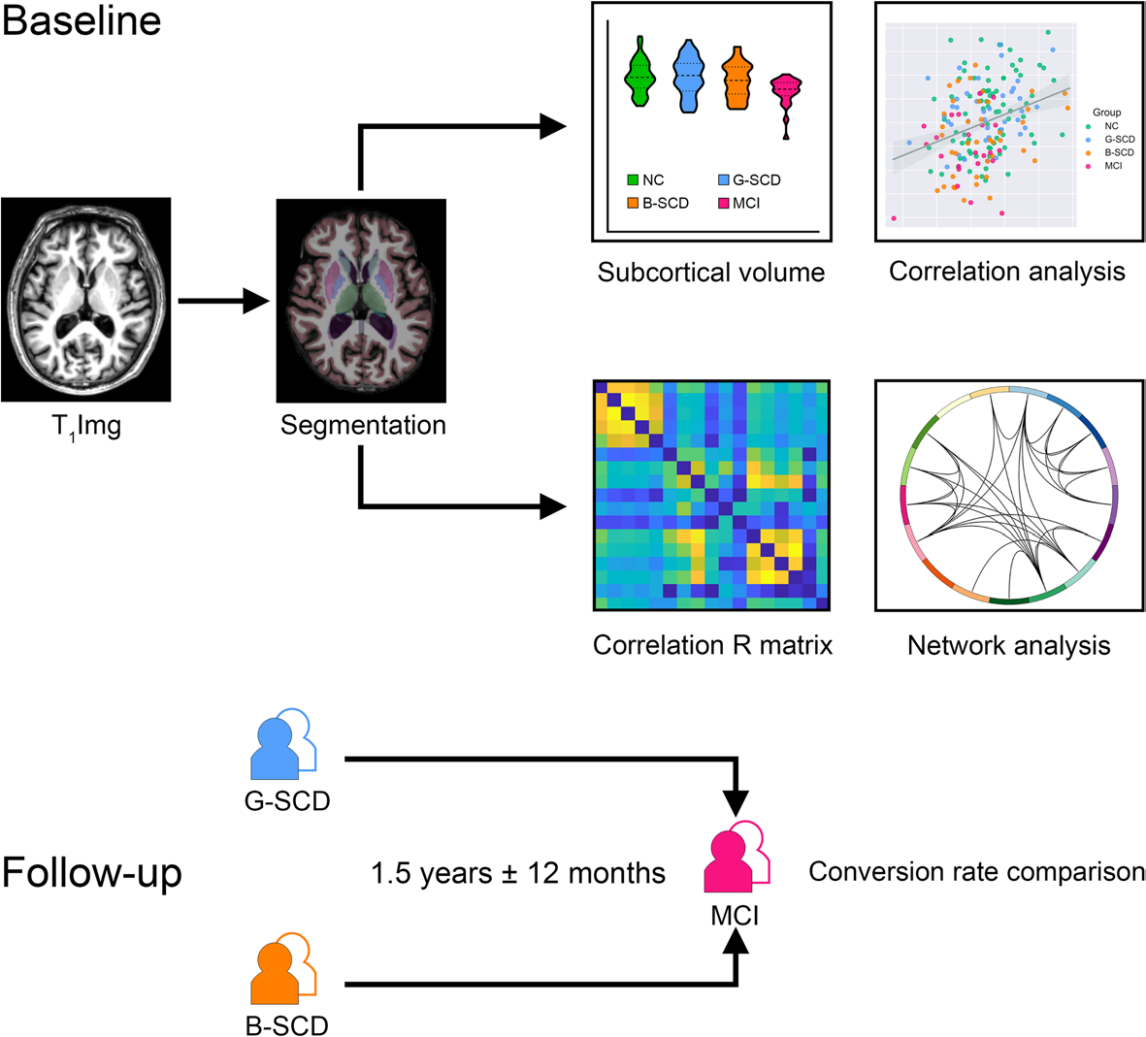
The flowchart of data processing and analysis steps

**Fig. 2:**
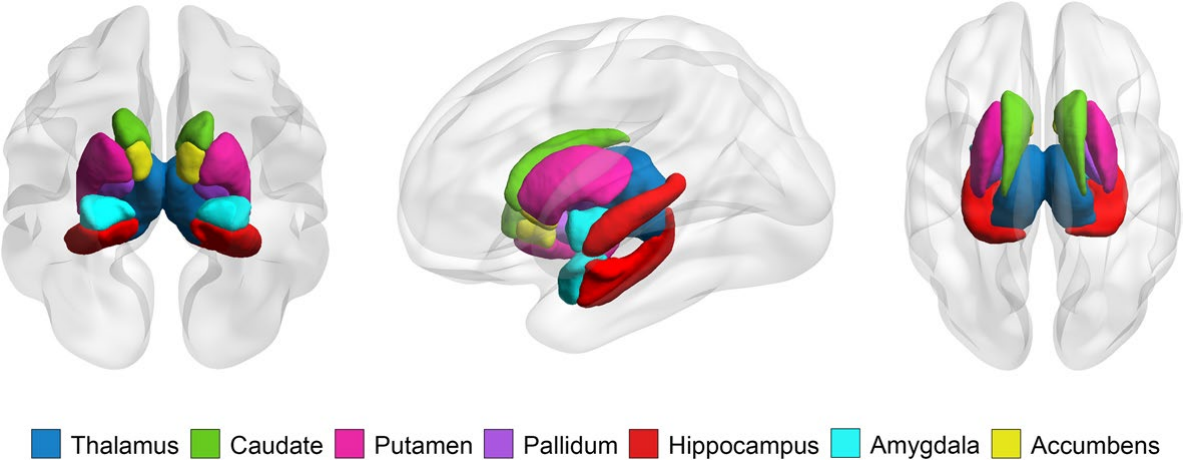
Anatomical position and extent of the subcortical structures

The flowchart of data processing and analysis steps was summarized in Fig. 1. The subcortical nuclei of thalamus, caudate, putamen, pallidum, hippocampus, amygdala, and accumbens were automatically segmented using the FreeSurfer version 6.0.0 image analysis suites (http://freesurfer.net/) (Fig. 2). The hippocampus was further divided into 12 subfields, including the hippocampal tail, subiculum, CA1, fissure, presubiculum, parasubiculum, molecular layer, dentate gyrus, CA2/3, CA4, fimbria, and HATA. In addition, the estimated total intracranial volume (TIV) was extracted to adjust for head size differences.

### Volume extraction of the basal forebrain subfields

**Fig. 3:**
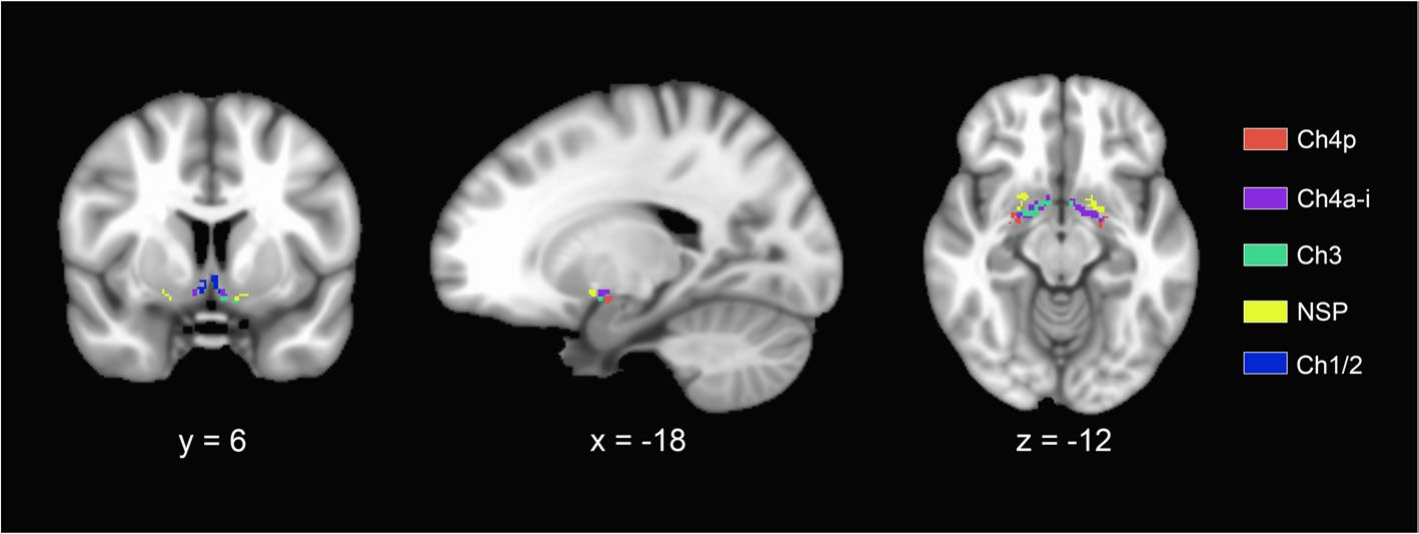
Segmentation of the basal forebrain subfields

A cytoarchitectonic mask in the Montreal Neurological Institute (MNI) space of the basal forebrain derived from histological sections of a postmortem brain was used to extract the basal forebrain volumes [53] (Fig. 3). Structural MRI data were processed using the Computational Anatomy Toolbox (CAT12, https://neuro-jena.github.io/cat/) based on Statistics Parametric Mapping version 12 (SPM12, https://www.fil.ion.ucl.ac.uk/spm/). Briefly, structural data were automatically segmented into gray matter, white matter, and cerebrospinal fluid partitions. Then, the grey matter was non-linearly normalized to the CAT12 default template (IXI555-MNI152) using the Diffeomorphic Anatomical Registration Through Exponentiated Lie Algebra (DARTEL) algorism. The images were modulated and smoothed with an 8-mm full-width at half-maximum (FWHM) [54, 55]. The weighted average image quality rating (IQR) of each participant was extracted from CAT12 to measure the image quality [56, 57].

The basal forebrain was divided into five subfields. Subfields of Ch4p (posterior part of the nucleus basalis of Meynert), Ch4a-i (anterior and intermediate parts of the nucleus basalis of Meynert), Ch3 (the nucleus of the horizontal limb of the diagonal band), NSP (the nucleus subputaminalis), and Ch1/2 (the nucleus of the vertical limb of the diagonal band) were derived. Volumes of all subfields were summed as the volume of the basal forebrain nuclei. Lower anatomical volumes indicate a more severe neural loss in the corresponding subcortical nuclei.

### Structural covariance network construction

The structural covariance network was constructed using the Brain Connectivity Toolbox (https://sites.google.com/site/bctnet/) [58]. Nodes were defined by the 12 subfields of the right hippocampus and 5 subfields of the basal forebrain. Edges were defined by the correlation coefficients between pairs of regional volumes of the nodes. Thus, a 17 × 17 R matrix for each group was generated. Considering that negative correlations were difficult to interpret, we set the negative correlation values to zero as suggested by previous studies [59, 60]. The network was further binarized within a sparsity range of 18%-50% and an increment of 1% to ensure robustness and comparability. The lower bound of the sparsity range was determined using the graph theory network analysis (GRETNA) toolbox (https://www.nitrc.org/projects/gretna/) [61].

Global metrics of clustering coefficient (Cp), characteristic path length (Lp), Gamma, Lambda, small worldness (Sigma), global efficiency, and local efficiency were calculated. Cp is defined as the average Cp (the number of edges that exist between neighbors) across all nodes in the network and can reflect the network segregation of the brain. Lp is defined as the average shortest path length between all pairs of nodes in the network and is commonly used to measure network integration of the brain. The Gamma is the normalized Cp and is calculated as Cp_real network_/Cp_random network_. Similarly, the Lambda is the normalized Lp and is calculated as Lp_real network_/Lp_random network_. A reduced Gamma and/or Lambda indicates a more random topology and enhanced information integration [39, 62]. The number of random networks was set to 100 [41, 59]. Sigma is calculated as Gamma/Lambda, and a Sigma greater than 1 suggests that the brain network has small-world properties. Global efficiency is inversely related to the average shortest path length and could reflect the information transfer capacity of the network. Local efficiency is defined as the average local efficiency (the degree of fault tolerance when the first neighbors were eliminated) across all nodes in the network [63].

Nodal metrics of nodal efficiency, betweenness centrality (the fraction of all shortest paths passing through a node), and degree (the number of connections that a node has with the rest of the network) were calculated.

### Follow-up data analysis

With an interval of a mean = 1.5 years ± 12 months of follow-up [64], the neuropsychological assessment was re-performed, and diagnoses were re-evaluated using the same criteria as baseline enrollment. The proportion of converters to MCI was compared between the G-SCD and B-SCD groups using Fisher’s exact test.

### Statistical analysis

The demographic and clinical data across the four groups were compared using one-way ANOVA, and the sex and MRI distribution were compared with chi-square tests. One-way ANCOVA was used to measure the across-group differences in subcortical nuclei volumes, controlling for sex, age, years of education, and TIV. Least significant difference post hoc was performed to compare each two groups. The correlations between subcortical nuclei volumes and clinical data were calculated, with sex, age, years of education, and TIV as covariates. Statistical analyses were performed with SPSS version 26.0, and the significance level was set at *p* < 0.05 with two-tailed tests. False discovery rate (FDR) correction was used for multiple comparison corrections.

The non-parametric permutation test with 1000 repetitions was applied to assess the across-group differences in structural covariance network properties, which were integrated over all selected ranges of sparsity values as the area under the curve (AUC). The significance level was set at *p* < 0.05 with two-tailed tests. The FDR correction was applied for multiple comparisons of nodal metrics.

### Supplementary analyses

We recalculated the basal forebrain subfield volumes with a 4-mm FWHM smoothing kernel to investigate whether the degree of smoothing was responsible for the differences among the four diagnostic groups. The correlations between basal forebrain subfield volumes with a 4-mm FWHM smoothing kernel and clinical data were recalculated, with sex, age, years of education, and TIV as covariates. Furthermore, we divided the SCD subjects into the G-SCD group (*n* = 40) and the B-SCD group (*n* = 40) based on their memory, language, and executive function, respectively, and then the comparisons of subcortical volumes and conversion rate to MCI between the two groups were repeated.

## Results

### Demographic and clinical data

As is shown in Table 1, no significant difference in age, sex, and years of education was observed among the four groups. The G-SCD, B-SCD, and MCI groups all showed worse self-perceived memory function than the NC group. The MCI group scored lower in the MMSE, memory, and executive cognitive domains than the other three groups. Compared to the G-SCD group, the B-SCD group showed worse language and executive function. The G-SCD group showed similar performance to the NC group on all the cognitive domains tested (Supplementary Fig. 1). The four groups did not significantly differ in TIV and IQR.

**Table 1:** Demographic and clinical data

	NC (*n* = 77)	G-SCD (*n* = 40)	B-SCD (*n* = 40)	MCI (*n* = 23)	*F/χ*^*2*^	*P*
Age	65.36 ± 5.80	64.40 ± 5.49	65.18 ± 5.90	65.87 ± 5.80	0.382	0.766
Sex (male/female)	14/63	7/33	7/33	4/19	0.015	1.000
**Education years**	12.86 ± 2.97	12.68 ± 2.64	11.79 ± 2.36	12.65 ± 3.61	1.272	0.286
MRI (TX/CX)	30/47	13/27	17/23	9/14	0.889	0.828
SCD-Q	3.90 ± 1.99	5.68 ± 1.29^a^	5.81 ± 1.25^b^	5.20 ± 2.07^c^	15.395	< 0.001*
MMSE	28.73 ± 1.19	28.75 ± 1.28	28.43 ± 1.53	27.26 ± 3.55^cef^	4.564	0.004*
Composite *Z* scores of each cognitive domain						
Memory function	0.29 ± 0.78	0.10 ± 0.71	− 0.11 ± 0.78^b^	− 0.97 ± 0.73^cef^	16.872	< 0.001*
Language function	0.21 ± 0.82	0.24 ± 0.63	− 0.31 ± 0.79^bd^	− 0.59 ± 0.70^ce^	9.931	< 0.001*
Executive function	0.10 ± 0.60	0.28 ± 0.64	− 0.15 ± 0.65^bd^	− 0.56 ± 0.63^cef^	10.284	< 0.001*
Navigation distance errors	− 0.28 ± 0.65	− 0.51 ± 0.27	0.80 ± 0.69^bd^	0.42 ± 1.03^cef^	35.129	< 0.001*
TIV	0.05 ± 0.99	0.11 ± 0.90	− 0.09 ± 1.07	− 0.21 ± 1.10	0.674	0.569
IQR (%)	84.50 ± 1.93	84.69 ± 1.79	84.59 ± 1.73	84.93 ± 1.46	0.373	0.773

Data were presented as means ± standard deviation or number. Statistics for sex and MRI were derived from the chi-square test, and statistics for other variables were derived from one-way ANOVA

*SCD-Q* Subjective cognitive decline questionnaire, *MMSE* Mini-mental state examination, *TIV* Total intracranial volume, *IQR* Image quality rating

^*^p < 0.05

^a~f^Post hoc analyses showed a significant difference between groups. ^a^NC vs G-SCD; ^b^NC vs B-SCD; ^c^NC vs MCI; ^d^G-SCD vs B-SCD; ^e^G-SCD vs MCI; ^f^B-SCD vs MCI

### Subcortical nuclei volumes

Table 2 shows the comparisons of the volumes of the 15 subcortical nuclei across the four groups. Significant differences in the volumes of the basal forebrain and the right hippocampus were observed. Specifically, the B-SCD and MCI groups showed lower volumes in the basal forebrain than the NC and G-SCD groups. The MCI group showed lower right hippocampal volume than the other three groups. No significant differences in the volumes of the bilateral thalamus, caudate, putamen, pallidum, amygdala, accumbens, and left hippocampus were shown.

**Table 2:** Subcortical nuclei volumes (mm^3^)

	NC (*n* = 77)	G-SCD (*n* = 40)	B-SCD (*n* = 40)	MCI (*n* = 23)	*F*	*P*
Basal forebrain	535.24 ± 40.62	539.47 ± 51.05	515.08 ± 42.47^bd^	499.95 ± 36.37^ce^	5.327	0.002*
L-Thalamus	5946.67 ± 563.92	5955.59 ± 650.65	5801.25 ± 580.15	5567.23 ± 414.48	2.262	0.083
L-Caudate	3077.73 ± 354.22	3176.93 ± 371.77	3065.99 ± 417.08	3084.21 ± 403.91	0.752	0.523
L-Putamen	4519.79 ± 448.30	4493.13 ± 518.10	4440.52 ± 554.15	4398.10 ± 347.17	0.224	0.879
L-Pallidum	1862.60 ± 240.92	1847.12 ± 219.02	1807.77 ± 254.06	1781.75 ± 262.65	0.396	0.756
L-Hippocampus	3577.23 ± 352.48	3576.78 ± 424.65	3484.67 ± 345.05	3312.63 ± 293.79	2.899	0.037
L-Amygdala	1359.26 ± 221.99	1281.10 ± 230.21	1301.93 ± 220.96	1232.48 ± 210.16	2.638	0.051
L-Accumbens	502.48 ± 87.68	493.19 ± 82.03	487.24 ± 77.49	448.04 ± 91.01	2.075	0.105
R-Thalamus	5813.13 ± 576.93	5757.73 ± 576.39	5667.74 ± 572.91	5498.96 ± 420.97	1.494	0.218
R-Caudate	3176.42 ± 349.43	3277.09 ± 376.46	3171.37 ± 415.60	3226.93 ± 493.72	0.879	0.453
R-Putamen	4503.70 ± 501.74	4494.81 ± 492.07	4466.18 ± 546.24	4406.74 ± 401.24	0.112	0.953
R-Pallidum	1757.02 ± 198.94	1784.53 ± 213.82	1754.38 ± 250.43	1702.58 ± 246.80	0.180	0.910
R-Hippocampus	3678.94 ± 379.55	3677.84 ± 450.97	3575.85 ± 407.17	3319.50 ± 345.14^cef^	4.693	0.004*
R-Amygdala	1534.93 ± 278.04	1484.69 ± 256.68	1474.44 ± 231.41	1358.29 ± 171.53	2.838	0.040
R-Accumbens	494.64 ± 81.59	489.63 ± 83.88	475.37 ± 66.99	454.74 ± 78.25	1.220	0.304

Data were presented as means ± standard deviation

*L* Left, *R* Right

^*^p < 0.05, FDR corrected, controlling for sex, age, years of education, and total intracranial volume

^a~f^Post hoc analyses showed a significant difference between groups. ^a^NC vs G-SCD; ^b^NC vs B-SCD; ^c^NC vs MCI; ^d^G-SCD vs B-SCD; ^e^G-SCD vs MCI; ^f^B-SCD vs MCI

Furthermore, subfields of the basal forebrain (Table 3) and the right hippocampus (Table 4) were analyzed. The B-SCD group showed lower volumes in the Ch4p and Ch4a-i subregions than the G-SCD group. The MCI group showed lower volumes in all the subregions of the basal forebrain. As for the right hippocampus, the MCI group showed atrophied subregions of the subiculum, CA1, presubiculum, molecular layer, dentate gyrus, CA4, and HATA.

**Table 3:** Basal forebrain subfield volumes (mm^3^)

	NC (*n* = 77)	G-SCD (*n* = 40)	B-SCD (*n* = 40)	MCI (*n* = 23)	*F*	*P*
Ch4p	85.80 ± 8.03	86.40 ± 9.27	81.96 ± 6.34^bd^	79.78 ± 5.74^ce^	4.994	0.002*
Ch4a-i	142.37 ± 10.17	144.01 ± 13.01	137.13 ± 11.32^bd^	134.19 ± 10.03^ce^	4.757	0.003*
Ch3	135.73 ± 10.25	136.77 ± 13.27	130.98 ± 11.29	126.73 ± 9.47^ce^	4.843	0.003*
NSP	104.60 ± 8.16	104.93 ± 10.01	101.44 ± 9.79	98.22 ± 7.23^ce^	3.187	0.025*
Ch1/2	66.74 ± 6.73	67.36 ± 8.00	63.56 ± 6.17	61.03 ± 6.91^ce^	4.885	0.003*

Data were presented as means ± standard deviation

^*^p < 0.05, FDR corrected, controlling for sex, age, years of education, and total intracranial volume

^a~f^Post hoc analyses showed a significant difference between groups. ^a^NC vs G-SCD; ^b^NC vs B-SCD; ^c^NC vs MCI; ^d^G-SCD vs B-SCD; ^e^G-SCD vs MCI; ^f^B-SCD vs MCI

**Table 4:** Right hippocampal subfield volumes (mm^3^)

	NC (*n* = 77)	G-SCD (*n* = 40)	B-SCD (*n* = 40)	MCI (*n* = 23)	*F*	*P*
Tail	533.30 ± 68.27	541.56 ± 73.41	521.76 ± 77.54	494.31 ± 68.98	1.651	0.180
Subiculum	405.03 ± 48.60	407.07 ± 50.87	398.36 ± 47.67	368.83 ± 47.03^cef^	3.168	0.026*
CA1	599.83 ± 77.12	590.84 ± 78.50	571.73 ± 69.33	539.58 ± 59.69^c^	3.776	0.012*
Fissure	174.85 ± 34.63	168.29 ± 29.36	164.64 ± 30.02	154.86 ± 24.73	2.287	0.080
Presubiculum	281.58 ± 29.56	278.40 ± 33.66	278.86 ± 30.81	256.79 ± 38.83^cf^	3.433	0.018*
Parasubiculum	52.84 ± 9.26	51.21 ± 8.47	52.46 ± 9.98	49.38 ± 7.39	0.917	0.434
Molecular layer	528.53 ± 58.90	525.75 ± 62.01	512.38 ± 53.09	474.96 ± 54.67^cef^	4.899	0.003*
Dentate gyrus	270.86 ± 34.67	264.55 ± 30.37	260.47 ± 27.93	242.87 ± 28.10^cef^	4.403	0.005*
CA2/3	185.85 ± 30.40	186.76 ± 28.44	179.64 ± 25.24	166.14 ± 25.92	2.611	0.053
CA4	229.72 ± 29.34	224.50 ± 25.27	222.68 ± 24.02	207.10 ± 22.62^cf^	3.974	0.009*
Fimbria	72.61 ± 17.86	64.33 ± 13.40^a^	65.69 ± 15.80^b^	64.57 ± 18.49	3.677	0.013*
HATA	55.43 ± 8.44	54.19 ± 9.15	53.15 ± 9.60	48.61 ± 8.14^c^	3.124	0.027*

Data were presented as means ± standard deviation

^*^p < 0.05, FDR corrected, controlling for sex, age, years of education, and total intracranial volume

^a~f^Post hoc analyses showed a significant difference between groups. ^a^NC vs G-SCD; ^b^NC vs B-SCD; ^c^NC vs MCI; ^d^G-SCD vs B-SCD; ^e^G-SCD vs MCI; ^f^B-SCD vs MCI

### Associations between subcortical nuclei volumes and clinical measures

**Fig. 4:**
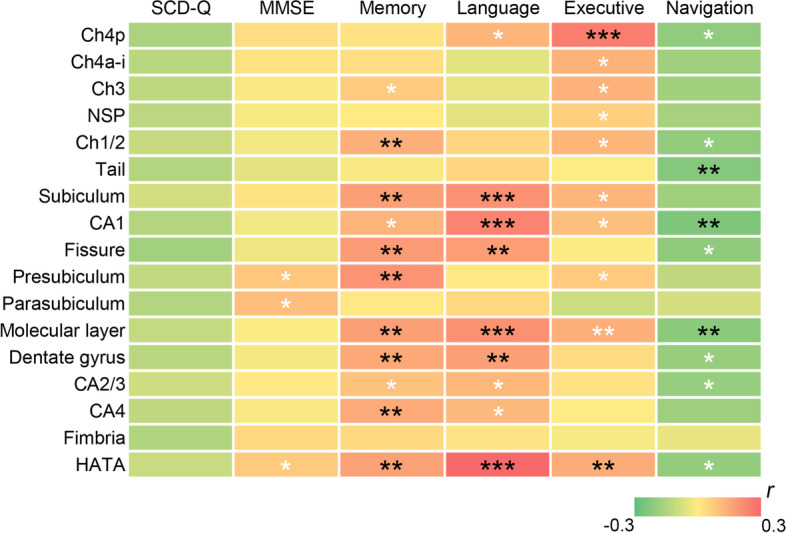
Associations between volumes of the basal forebrain and right hippocampal subfields and clinical measures. Partial correlation analyses were adjusted for sex, age, years of education, and total intracranial volume. *p < 0.05; **p < 0.01; ***p < 0.001. The black * indicates results that survived multiple comparisons after FDR correction

As is shown in Fig. 4, significant correlations between volumes of the basal forebrain and right hippocampal subfields and clinical measures were observed. After FDR correction, greater volumes in the Ch4p were associated with greater executive function (*r* = 0.271, *p* < 0.001). The volumes of right hippocampal subfields were significantly correlated with memory function (subiculum, *r* = 0.218, *p* = 0.004; fissure, *r* = 0.226, *p* = 0.003; presubiculum, *r* = 0.237, *p* = 0.002; molecular layer, *r* = 0.220, *p* = 0.003; dentate gyrus, *r* = 0.207, *p* = 0.006; CA4, *r* = 0.203, *p* = 0.007; HATA, *r* = 0.217, *p* = 0.004). The volumes of right hippocampal subfields were also significantly correlated with navigation function (tail, *r* =  − 0.210, *p* = 0.005; CA1, *r* =  − 0.227, *p* = 0.002; molecular layer, *r* =  − 0.196, *p* = 0.009). Under uncorrected criteria, more significant correlations were observed. Greater volumes in the Ch4p were associated with greater language (*r* = 0.186, *p* = 0.014) and navigation function (*r* =  − 0.180, *p* = 0.017). Greater volumes in the Ch4a-i were associated with great performance on the executive tests (*r* = 0.192, *p* = 0.011).

### Structural covariance network properties

**Fig. 5:**
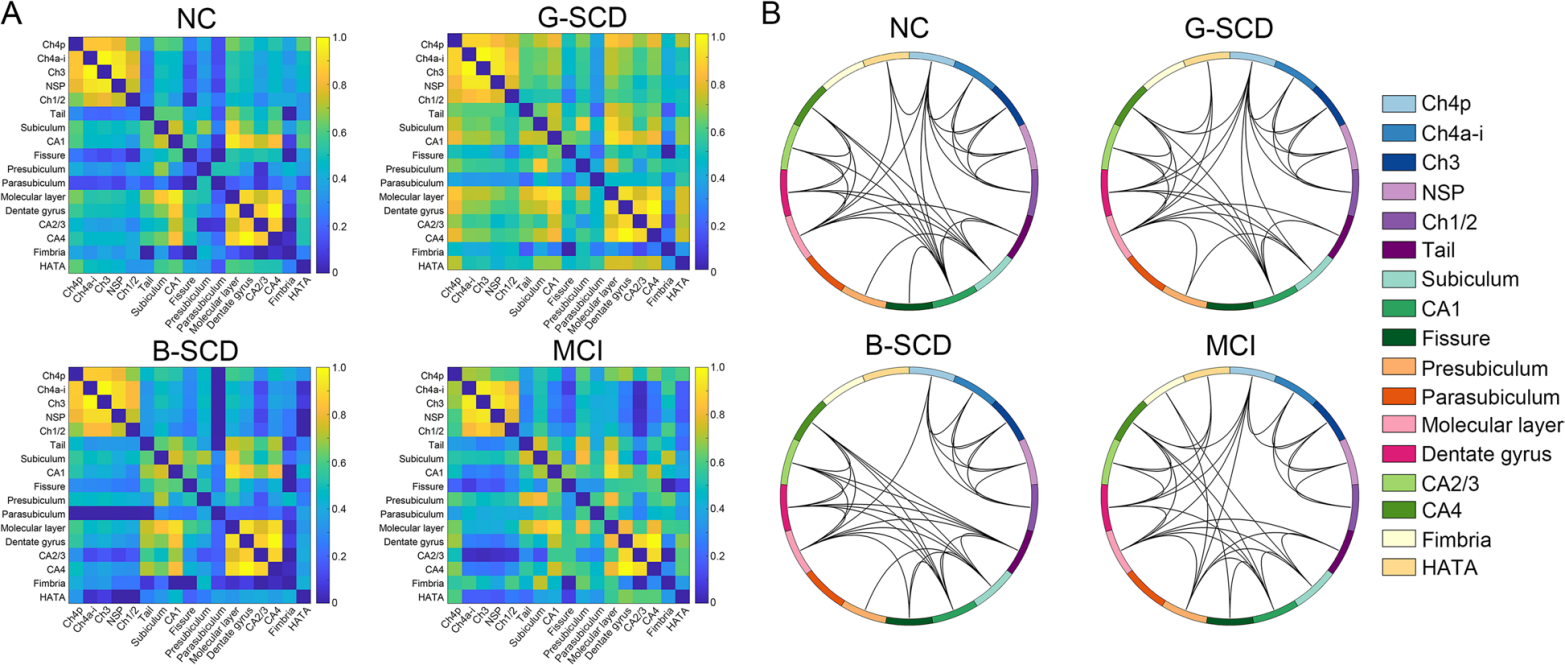
Structural covariance network of the basal forebrain and right hippocampal subfields. **A** Correlation R matrices of subfields in each diagnostic group. **B** The top 25% of the strongest regional connections are shown for each diagnostic group

Topological properties of the structural covariance network constructed by the subfields of the basal forebrain and the right hippocampus were assessed. The correlation matrix of each group is shown in Fig. 5A, and the 25% strongest connections of each diagnostic group are shown in Fig. 5B. Compared with the G-SCD group, the B-SCD group showed a larger Lambda (Table 5). The MCI group showed a larger Gamma than the G-SCD group. The MCI group also showed a lower Lp, a larger Sigma, and greater global efficiency than both G-SCD and B-SCD groups. Considering the nodal metrics, the MCI group showed greater nodal efficiency in the right fimbria than the G-SCD group.

**Table 5:** Global metrics of the structural covariance network

Global metrics	Group comparisons								
	NC	G-SCD	*p*	NC	B-SCD	*p*	NC	MCI	*p*
aCp	0.21	0.21	0.662	0.21	0.21	0.883	0.21	0.22	0.578
aLp	0.62	0.64	0.586	0.62	0.73	0.106	0.62	0.55	0.216
aGamma	0.50	0.43	0.366	0.50	0.54	0.659	0.50	0.61	0.301
aLambda	0.33	0.32	0.347	0.33	0.36	0.327	0.33	0.33	0.924
aSigma	0.48	0.42	0.141	0.48	0.47	0.804	0.48	0.59	0.090
aEglobal	0.17	0.16	0.314	0.17	0.16	0.226	0.17	0.19	0.102
aElocal	0.23	0.23	0.635	0.23	0.23	0.852	0.23	0.25	0.138
Global metrics	Group comparisons								
	G-SCD	B-SCD	*p*	G-SCD	MCI	*p*	B-SCD	MCI	*p*
aCp	0.21	0.21	0.810	0.21	0.22	0.797	0.21	0.22	0.717
aLp	0.64	0.73	0.079	0.64	0.55	0.034*	0.73	0.55	0.005*
aGamma	0.43	0.54	0.154	0.43	0.61	0.009*	0.54	0.62	0.482
aLambda	0.32	0.36	0.021*	0.32	0.33	0.090	0.36	0.33	0.531
aSigma	0.42	0.47	0.163	0.42	0.59	< 0.001*	0.47	0.59	0.012*
aEglobal	0.16	0.16	0.718	0.16	0.19	0.012*	0.16	0.19	0.005*
aElocal	0.23	0.23	0.809	0.23	0.25	0.164	0.23	0.25	0.226

*aCp* area under the curve (AUC) of the clustering coefficient, *aLp* AUC of the shortest path length, *aGamma* AUC of the Gamma, *aLambda* AUC of the Lambda, *aSigma* AUC of the Sigma, *aEglobal*, AUC of the global efficiency, *aElocal* AUC of the local efficiency

^*^p < 0.05

### Follow-up data analytical results

With an interval of a mean = 1.5 years ± 12 months of follow-up, none in the G-SCD group (*n* = 20) progressed to MCI, while 4 in the B-SCD group (*n* = 19) progressed to MCI, with a follow-up interval of 396, 723, 761, and 791 days, respectively. The G-SCD and B-SCD groups differed significantly in the conversion rate to MCI. No significant difference in interval days and demographic data of the follow-up cohort was shown between the two groups (Table 6).

**Table 6:** Follow-up data

	G-SCD (*n* = 20)	B-SCD (*n* = 19)	*t*	*P*
Age	65.50 ± 5.51	65.16 ± 6.05	0.185	0.854
Sex (male/female)	5/15	3/16		0.695
Education	12.85 ± 2.81	11.71 ± 2.35	1.368	0.180
Outcome (converters/nonconverters)	0/20	4/15		0.047*
Interval (days)	535.95 ± 163.50	539.16 ± 168.55	− 0.060	0.952

Data were presented as means ± standard deviation or number. The *p* values for sex and outcome were derived from Fisher’s exact test, and statistics for other variables were derived from two sample *t*-test

^*^p < 0.05

### Supplementary analytical results

We smoothed the modulated images with a 4-mm instead of 8-mm FWHM, and the result of lower volumes in the Ch4p and Ch4a-i subregions in the B-SCD group than the G-SCD group remained robust (Supplementary Table 1), which suggested that the degree of smoothing may not be a crucial factor for the differences. The correlations between basal forebrain subfield volumes with a 4-mm FWHM smoothing kernel and clinical measures were shown in Supplementary Fig. 2. After FDR correction, greater volumes in the Ch4p were associated with greater executive function (*r* = 0.250, *p* < 0.001). Under uncorrected criteria, greater volumes in the Ch4p were associated with greater language (*r* = 0.162, *p* = 0.032) and navigation function (*r* =  − 0.168, *p* = 0.026).

In addition, the SCD subjects were divided into the G-SCD group (*n* = 40) and the B-SCD group (*n* = 40) based on their memory, language, and executive function, respectively. Contrary to the reduced volumes in Ch4p and Ch4a-i subfields of the basal forebrain in the B-SCD than the G-SCD grouped by the navigation ability, no significant differences in volumes of basal forebrain subfields were observed between the G-SCD and the B-SCD grouped by their memory, language, and executive function (Supplementary Table 2). Furthermore, contrary to the higher conversion rate to MCI in the B-SCD than the G-SCD grouped by the navigation ability, no significant differences in conversion rate to MCI were observed between the G-SCD and the B-SCD grouped by their memory (Supplementary Table 3), language (Supplementary Table 4), and executive function (Supplementary Table 5).

## Discussion

In the present study, for the first time, we investigated the heterogeneity of SCD from the perspective of spatial navigation performance. The SCD participants were divided into two subgroups based on their spatial navigation performance. The results showed that, compared to SCD subjects with good spatial navigation ability, those with bad spatial navigation ability showed lower volumes in the basal forebrain, reorganized structural covariance network of the basal forebrain and right hippocampal subfields, and a higher conversion rate to MCI. Altogether, this study may indicate the promising role of spatial navigation in risk assessment and early intervention for SCD subjects.

It has been well-established by histopathological studies that AD is associated with the loss of cholinergic neurons and the basal forebrain is a key structure for cholinergic input to the hippocampus, amygdala, and cerebral cortex [65, 66]. Cholinergic degeneration in the basal forebrain plays a crucial role during AD progression, not only in late disease but also in the early stages [67, 68]. Significant associations between basal forebrain atrophy and cortical amyloid deposition in patients with pre-symptomatic and predementia stages of AD have been observed [55]. In addition, degeneration of the basal forebrain cholinergic projections has been suggested as a robust and reliable upstream event of entorhinal and neocortical degeneration [64]. Previous studies have shown significant volume reductions of the basal forebrain in SCD, MCI, and AD patients [24, 29, 69]. In accordance with previous studies, the B-SCD group and MCI group in this study showed lower volumes in the basal forebrain, while this was not observed in the G-SCD group. Contrary to the significantly atrophied right hippocampus observed in the MCI group, no significant hippocampal atrophy was observed in the B-SCD group. These findings are aligned with previous studies suggesting that volume reduction in the basal forebrain may be a more sensitive structural indicator than hippocampal atrophy in the early stages of AD [70, 71]. These findings also indicate that treatment with acetylcholinesterase inhibitors may be noteworthy since the earliest stages of AD.

Subfield and correlation analyses showed that the B-SCD group mainly showed atrophy in the Ch4p and Ch4a-i subfields of the basal forebrain and the volumes of these subfields were significantly associated with language and executive function as well as with spatial navigation performance under uncorrected criteria. Cholinergic inputs to the cerebrum originate from different nuclei of the basal forebrain, with Ch1 and Ch2 innervating the hippocampus, Ch3 innervating the olfactory bulb, and Ch4 innervating practically the entire cerebral cortex and amygdala [72, 73]. The Ch4, also known as the nucleus basalis of Meynert, is a key structure for cholinergic input to the medial prefrontal, cingulate, retrosplenial, and visual cortices [74]. The medial prefrontal lobe plays a crucial role in many aspects of navigation, including route planning, route plan updating, goal tracking, path selection, and spatial memory consolidation and abstraction [75]. The posterior cingulate and retrosplenial cortex, which are located close to both the medial temporal and parietal lobes, have been implicated in the integration of hippocampal-related allocentric and parietal-related egocentric spatial information and the flexible transitions between these two navigation strategies [27, 76]. We speculated that the atrophy in Ch4 may trigger a loss of cholinergic axons projecting to the associative cortical regions and eventually leads to cognitive and spatial navigation deficits. Notably, the correlations between Ch4p volumes and navigation performance did not survive multiple comparisons. We speculated that the behavioral deficits in the B-SCD group are more likely to be underlined by functional rather than structural neural changes, which would be further investigated in our future study.

Except for the subcortical volumetry, we also studied the organization of the structural covariance network. Since significant differences in the volumes of the right hippocampus rather than the left hippocampus among the diagnostic groups were observed and hippocampal lateralization in navigation has been suggested by previous studies [13, 77], thus, only the right hippocampal subfields were used to construct the structural covariance network combined with basal forebrain subfields. The findings showed that the B-SCD group had a larger Lambda than the G-SCD group, suggesting a reorganized structural covariance and weakened network integration of the basal forebrain and right hippocampal subfields. Fu et al. have observed an altered organization of the grey matter network characterized by decreased properties of integration and segregation in SCD individuals compared to the NCs [41]. Significant associations between global amyloid burden and individual gray matter network properties in participants with subjective memory complaints have been reported [78]. In addition, alterations in individual grey matter network measures could predict faster clinical progression in subjects with SCD or MCI [39, 40]. These studies suggested that a more random organization of the grey matter network was associated with a steeper cognitive decline, with a lower Lambda predicting a steeper decline in global cognition and language function. This finding may somewhat be inconsistent with the larger Lambda and higher conversion rate to MCI in the B-SCD group in the present study. Possible explanations for this discrepancy could be the differences in the SCD definition, participant recruitment, and analytical methods of structural covariance network, e.g., individual or group-wise, the definition of network nodes, sparsity, and other parameters. The MCI group in the present study showed an increased small-word property compared to the SCD group. Consistent with this, a recent study conducted on 194 elderly subjects with records on amyloid and tau status also suggested that the structural covariance was enhanced during AD progression [79]. Increased structural covariance seeding from hippocampal subfields and basal forebrain in MCI patients has been reported by the voxel-based analysis [80, 81]. We speculated that the parallel pattern of the basal forebrain and hippocampal atrophy may contribute to the enhanced structural covariance in the MCI stage. The abnormalities of morphometric networks, which seem divergent and dynamic across the different stages of AD, may contribute to our understanding of the neuropathological mechanisms.

Recently, researchers who focus on SCD have been emphasizing that the heterogeneity of this entity needs to be further investigated [8–11, 82, 83]. In the present study, for the first time, we investigated the heterogeneity of SCD from the perspective of spatial navigation performance. The neural structural basis underlying spatial navigation (e.g., basal forebrain and hippocampus) are affected by AD pathology since the earliest stages [26]. Furthermore, compared to the traditional paper-and-pencil-based cognitive tests, the spatial navigation tests have relatively fewer verbal, cultural, and educational biases, which may facilitate cross-cultural clinical trials across different sites [16]. Previous longitudinal studies have demonstrated the promising utility of spatial navigation at baseline as an assessment tool in predicting future cognitive decline. In a prospective cohort study of 442 non-demented adults with a mean follow-up of 16.5 ± 13.7 months, Verghese et al. found that a 10-s increment on the immediate maze time measured by the Floor Maze Test could predict the incidence of MCI with an adjusted hazard ratio of 1.25 [20]. Levine et al. assessed the diagnostic value of cognitive mapping, route learning, and episodic memory tests in predicting clinical progression over an average of 4–5 years, and the findings showed an AUC of 0.894, 0.794, and 0.735, respectively; the cognitive mapping tended to perform better than the episodic memory [19]. Consistent with these findings, our follow-up data showed that the B-SCD group had a significantly higher conversion rate to MCI than the G-SCD group. Our findings indicated that spatial navigation may have great potential to investigate the heterogeneity of SCD and to identify SCD subjects at higher risk of clinical progression. Supplementary analytical results showed that no significant differences in volumes of basal forebrain subfields and conversion rate to MCI were observed between the G-SCD and B-SCD grouped by their memory, language, or executive function, which may further support that spatial navigation could serve as a more informative and promising tool for risk assessment and early intervention for SCD.

## Limitations

This study has some limitations. First, the sample size was relatively small and follow-up data was not available for all SCD participants. Second, AD biomarkers of amyloid β and tau were not available; thus, this study could not provide direct evidence on whether baseline spatial navigation could help identify the SCD subjects with positive AD biomarkers from the whole SCD entity. Recent studies have indicated the promising utility of plasma AD biomarkers, which are easier to obtain [84, 85]. We will collect plasma AD biomarkers in our future work and try to investigate whether spatial navigation could also help identify SCD with positive AD biomarkers or SCD at the preclinical stage of AD. Third, only structural imaging data were analyzed, while multimodal imaging combined with functional MRI and diffusion tensor imaging may give a more comprehensive description of the differences in imaging markers between the G-SCD and B-SCD groups. Fourth, the spatial navigation test was based on a two-dimensional computerized paradigm, while those based on real space and virtual reality may be more accurate. Lastly, the relatively large variation in the follow-up period may have an impact on the conversion rate, and the follow-up duration may not be long enough to record the final outcome of the SCD participants to determine a prognostic model for SCD based on the present data. The development of a prognostic model for SCD based on combined features of demographics, cognition, spatial navigation, plasma biomarkers, and imaging markers in the baseline will be our focus in the future.

## Conclusions

Compared to SCD subjects with good spatial navigation performance, SCD subjects with bad performance showed lower volumes in the basal forebrain, a reorganized structural covariance network of subcortical nuclei, and an increased risk of progression to MCI. Our findings may provide new insights into the role of spatial navigation in identifying those with SCD who are at higher risk of clinical progression to objective cognitive impairment, which may contribute to making more precise clinical decisions for SCD individuals who seek medical help.

## Supplementary Information

**Additional file 1: Supplementary Fig. 1.** Cognitive and spatial navigation performance among the four diagnostic groups. *, *p* < 0.05. **Supplementary Fig. 2.** Associations between volumes of the basal forebrain using a 4-mm smoothing kernel and right hippocampal subfields and clinical measures. Partial correlation analyses were adjusted for sex, age, years of education, and total intracranial volume. *, *p* < 0.05; **, *p* < 0.01; ***, *p* < 0.001. The black * indicates results that survived multiple comparisons after FDR correction.

**Additional file 2: Supplementary Table 1.** Basal forebrain subfield volumes with a 4-mm FWHM smoothing kernel.

**Additional file 3: Supplementary Table 2.** Basal forebrain subfield volumes based on different grouping methods of SCD.

**Additional file 4: Supplementary Table 3.** Follow-up data grouped by memory function.

**Additional file 5: Supplementary Table 4.** Follow-up data grouped by language function.

**Additional file 6: Supplementary Table 5.** Follow-up data grouped by executive function.

## Data Availability

Due to the clinical nature of the data, the data that support the findings of this study are not freely available but can be made available by the corresponding author, upon reasonable request. A formal data-sharing agreement is needed before any data can be shared.

## Abbreviations

AD: Alzheimer’s disease
SCD: Subjective cognitive decline
MCI: Mild cognitive impairment
NC: Normal control
T_1_W: T_1_-weighted
AAL: Automated Anatomical Labelling
MRI: Magnetic resonance imaging
SILCODE: Sino Longitudinal Study on Cognitive Decline
SCD-Q: SCD-questionnaire
MMSE: Mini-mental state examination
AVLT: Auditory verbal learning test
TMT-A: Trail making test part A
TMT-B: Trail making test part B
SDMT: Symbol digit modalities test
CDT: Clock drawing test
AFT: Animal fluency test
BNT: Boston naming test
G-SCD: SCD-good
B-SCD: SCD-bad
TR: Repetition time
TE: Echo time
FOV: Field of view
TIV: Total intracranial volume
MNI: Montreal Neurological Institute
DARTEL: Diffeomorphic Anatomical Registration Through Exponentiated Lie Algebra
FWHM: Full-width at half-maximum
IQR: Image quality rating
Cp: Clustering coefficient
Lp: Characteristic path length
Sigma: Small worldness
FDR: False discovery rate
AUC: Area under the curve
