# Supplementary material for: Spatial navigation is associated with subcortical alterations and progression risk in subjective cognitive decline
Source: Alzheimers Res Ther. 2023 Apr 25;15:86. doi: 10.1186/s13195-023-01233-6 (PMC10127414; doi:10.1186/s13195-023-01233-6)
Supplement: Supplementary file 1 — Additional file 1: Supplementary Fig. 1. Cognitive and spatial navigation performance among the four diagnostic groups. *, p < 0.05. Supplementary Fig. 2. Associations between volumes of the basal forebrain using a 4-mm smoothing kernel and right hippocampal subfields and clinical measures. Partial correlation analyses were adjusted for sex, age, years of education, and total intracranial volume. *, p < 0.05; **, p < 0.01; ***, p < 0.001. The black * indicates results that survived multiple comparisons after FDR correction. [file 13195_2023_1233_MOESM1_ESM.zip › Supplementary_materials.docx]

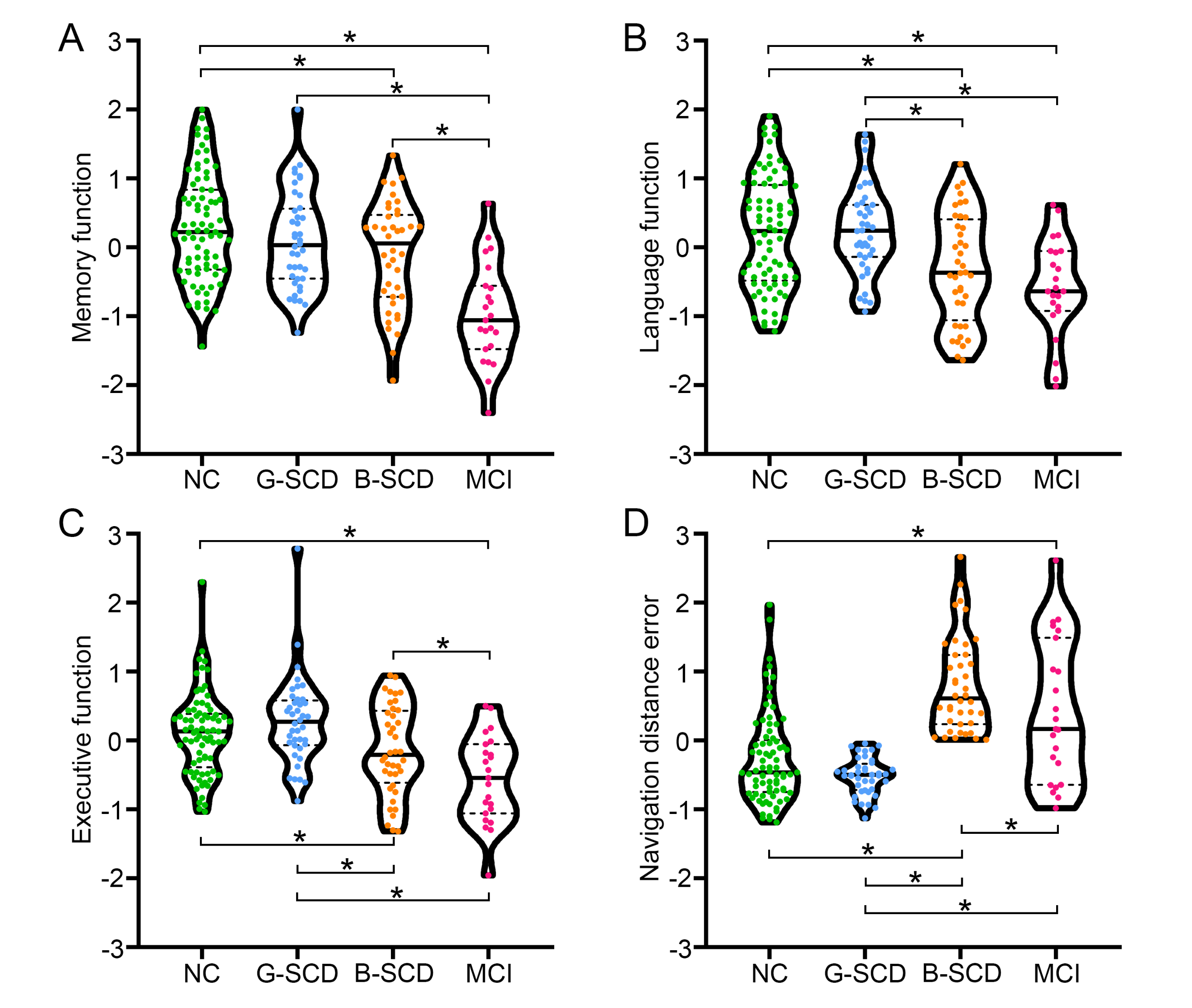


**Supplementary Fig. 1** Cognitive and spatial navigation performance among the four diagnostic groups. *, *p* < 0.05.


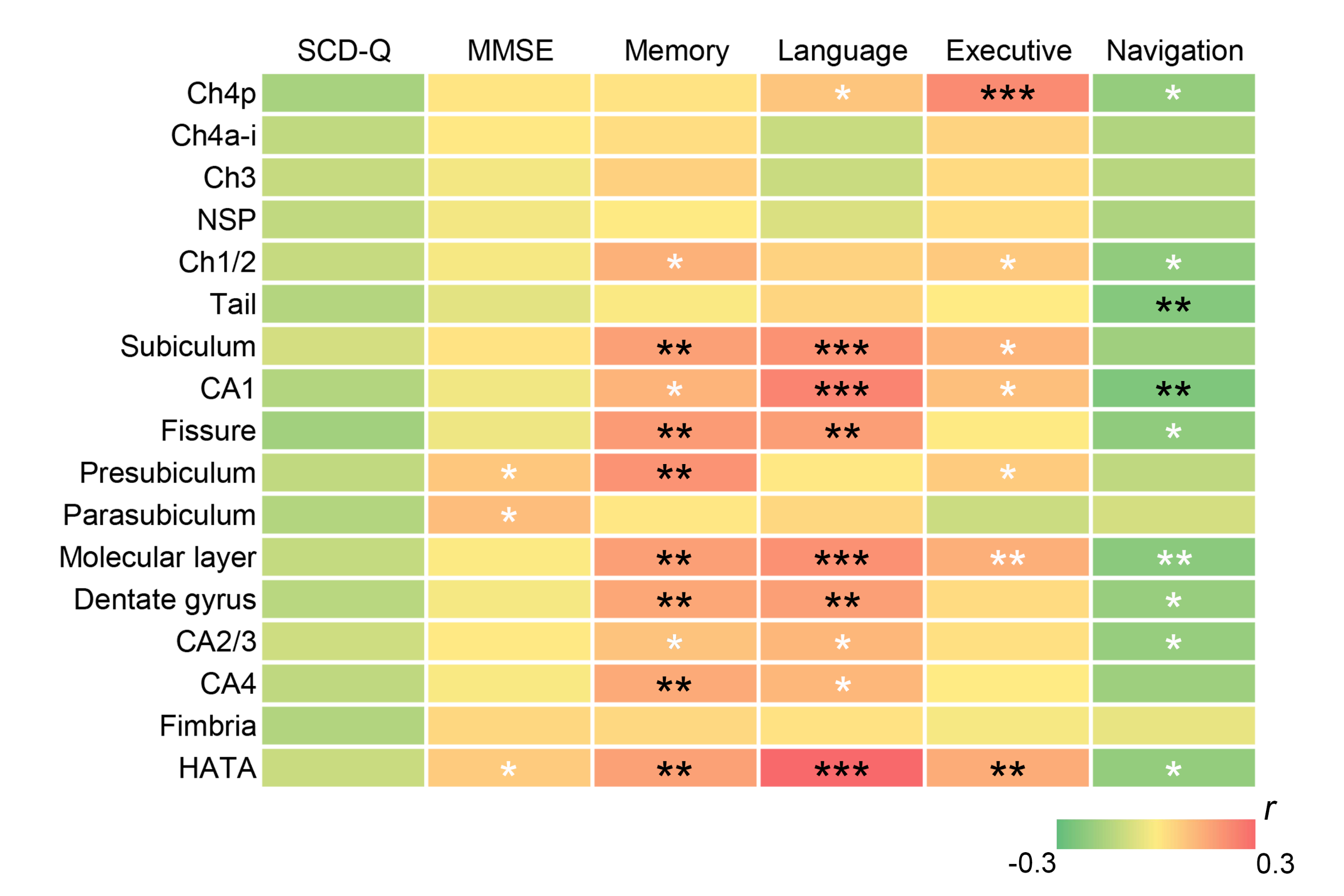


**Supplementary Fig. 2** Associations between volumes of the basal forebrain using a 4-mm smoothing kernel and right hippocampal subfields and clinical measures. Partial correlation analyses were adjusted for sex, age, years of education, and total intracranial volume. *, *p* < 0.05; **, *p* < 0.01; ***, *p* < 0.001. The black * indicates results that survived multiple comparisons after FDR correction.

**Supplementary Table 1 Basal forebrain subfield volumes with a 4-mm FWHM smoothing kernel**

|  | NC  (n = 77) | G-SCD  (n = 40) | B-SCD  (n = 40) | MCI  (n = 23) | *F* | *P* |
| --- | --- | --- | --- | --- | --- | --- |
| Ch4p | 97.47±9.53 | 98.04±11.48 | 92.60±7.43^bd^ | 90.51±6.48^ce^ | 4.904 | 0.003* |
| Ch4a-i | 150.91±10.74 | 153.16±14.32 | 145.84±13.56^d^ | 142.21±11.28^ce^ | 4.200 | 0.007* |
| Ch3 | 148.24±11.50 | 150.40±15.19 | 143.67±14.43 | 138.66±12.10^ce^ | 3.877 | 0.010* |
| NSP | 117.22±9.56 | 117.94±12.21 | 113.78±11.52 | 109.58±7.89^ce^ | 3.282 | 0.022* |
| Ch1/2 | 75.94±7.96 | 76.69±9.37 | 72.26±7.31 | 69.09±8.11^ce^ | 4.951 | 0.003* |

Data were presented as means±standard deviation. *, *p* < 0.05, FDR corrected, controlling for sex, age, years of education, and total intracranial volume. ^a~f^, post hoc analyses showed a significant difference between groups. ^a^: NC vs G-SCD; ^b^: NC vs B-SCD; ^c^: NC vs MCI; ^d^: G-SCD vs B-SCD; ^e^: G-SCD vs MCI; ^f^: B-SCD vs MCI.

**Supplementary Table 2 Basal forebrain subfield volumes based on different grouping methods of SCD**

|  | Group by navigation ability | |  | Group by memory function | |  | Group by language function | |  | Group by executive function | |
| --- | --- | --- | --- | --- | --- | --- | --- | --- | --- | --- | --- |
|  | G-SCD  (n = 40) | B-SCD  (n = 40) |  | G-SCD  (n = 40) | B-SCD  (n = 40) |  | G-SCD  (n = 40) | B-SCD  (n = 40) |  | G-SCD  (n = 40) | B-SCD  (n = 40) |
| Ch4p | 86.40±9.27 | 81.96±6.34* |  | 84.85±8.85 | 83.51±7.55 |  | 84.95±8.71 | 83.41±7.70 |  | 85.79±7.48 | 82.57±8.66 |
| Ch4a-i | 144.01±13.01 | 137.13±11.32* |  | 141.37±13.39 | 139.78±11.88 |  | 140.15±13.55 | 141.00±11.74 |  | 142.86±11.19 | 138.29±13.64 |
| Ch3 | 136.77±13.27 | 130.98±11.29 |  | 134.70±13.20 | 133.04±12.05 |  | 133.21±13.42 | 134.54±11.82 |  | 136.04±10.85 | 131.71±13.90 |
| NSP | 104.93±10.01 | 101.44±9.79 |  | 103.48±10.34 | 102.89±9.75 |  | 102.66±10.07 | 103.71±10.01 |  | 104.03±9.00 | 102.33±10.94 |
| Ch1/2 | 67.36±8.00 | 63.56±6.17 |  | 66.56±7.85 | 64.37±6.74 |  | 65.81±8.49 | 65.11±6.10 |  | 66.47±6.61 | 64.45±7.98 |

Data were presented as means±standard deviation. *, *p* < 0.05, controlling for sex, age, years of education, and total intracranial volume.

**Supplementary Table 3 Follow-up data grouped by memory function**

| Group by memory function | G-SCD  (n = 16) | B-SCD  (n = 23) | *t* | *P* |
| --- | --- | --- | --- | --- |
| Age | 64.31±5.17 | 66.04±6.06 | -0.930 | 0.358 |
| Sex (Male/Female) | 2/14 | 6/17 |  | 0.432 |
| Education | 12.31±2.82 | 12.28±2.55 | 0.034 | 0.973 |
| Outcome (converters/nonconverters) | 0/16 | 4/19 |  | 0.130 |
| Interval (days) | 479.38±128.07 | 577.96±176.02 | -1.912 | 0.064 |

Data were presented as means±standard deviation or number. The *p* values for sex and outcome were derived from Fisher's exact test, and statistics for other variables were derived from two sample t-test.

**Supplementary Table 4 Follow-up data grouped by language function**

| Group by language function | G-SCD  (n = 19) | B-SCD  (n = 20) | *t* | *P* |
| --- | --- | --- | --- | --- |
| Age | 64.68±6.37 | 65.95±5.08 | -0.688 | 0.496 |
| Sex (Male/Female) | 5/14 | 3/17 |  | 0.451 |
| Education | 13.05±2.20 | 11.58±2.85 | 1.805 | 0.079 |
| Outcome (converters/nonconverters) | 0/19 | 4/16 |  | 0.106 |
| Interval (days) | 536.47±151.30 | 538.50±178.78 | -0.038 | 0.970 |

Data were presented as means±standard deviation or number. The *p* values for sex and outcome were derived from Fisher's exact test, and statistics for other variables were derived from two sample t-test.

**Supplementary Table 5 Follow-up data grouped by executive function**

| Group by executive function | G-SCD  (n = 21) | B-SCD  (n = 18) | *t* | *P* |
| --- | --- | --- | --- | --- |
| Age | 64.19±5.77 | 66.67±5.49 | -1.367 | 0.180 |
| Sex (Male/Female) | 5/16 | 3/15 |  | 0.702 |
| Education | 12.91±2.55 | 11.58±2.61 | 1.596 | 0.119 |
| Outcome (converters/nonconverters) | 1/20 | 3/15 |  | 0.318 |
| Interval (days) | 525.19±158.10 | 551.89±173.64 | -0.502 | 0.618 |

Data were presented as means±standard deviation or number. The *p* values for sex and outcome were derived from Fisher's exact test, and statistics for other variables were derived from two sample t-test.
