# Supplementary figures and images for: Spatial navigation is associated with subcortical alterations and progression risk in subjective cognitive decline
Source: Alzheimers Res Ther. 2023 Apr 25;15:86. doi: 10.1186/s13195-023-01233-6 (PMC10127414; doi:10.1186/s13195-023-01233-6)

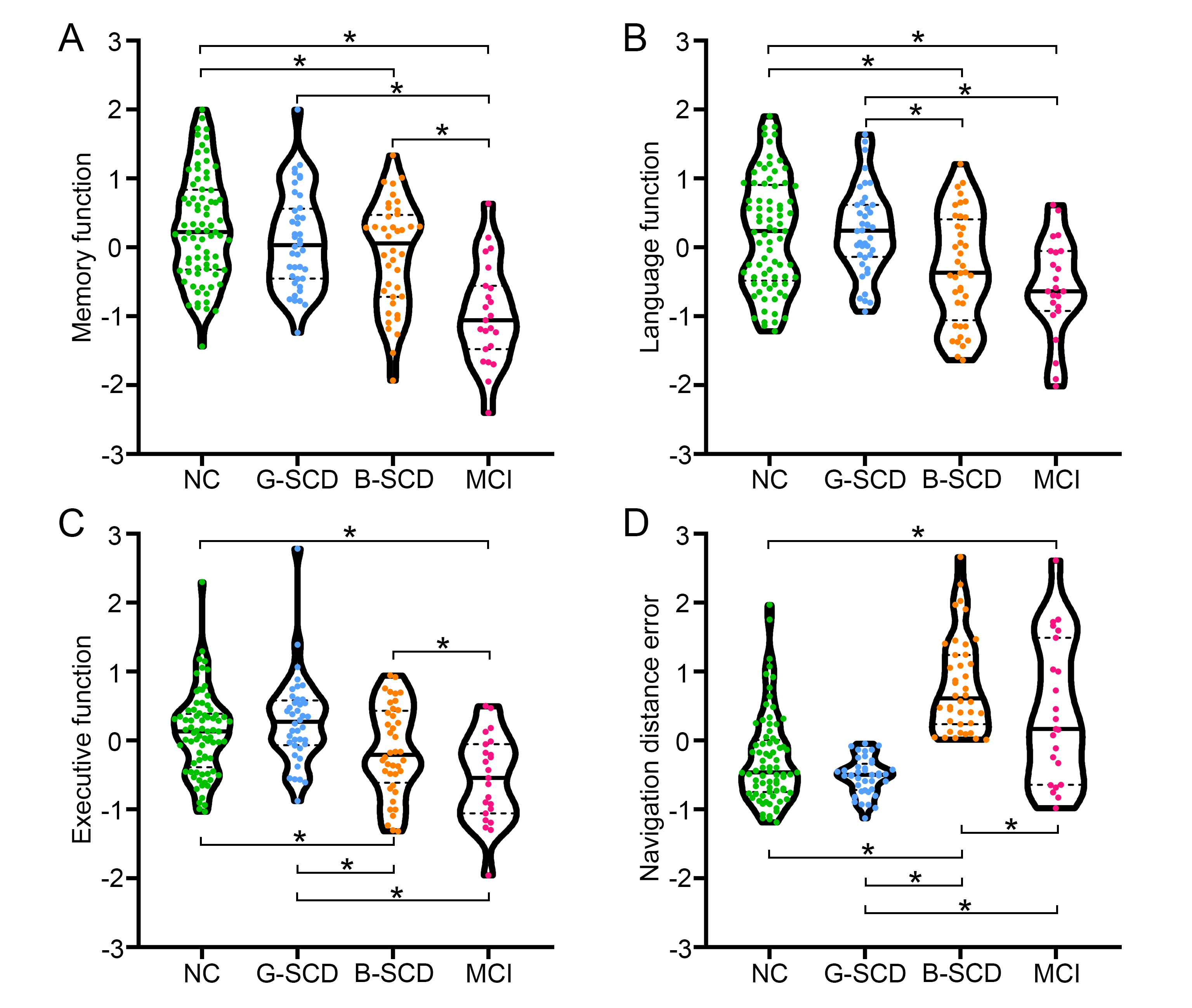

Supplement: Supplementary file 1 — Additional file 1: Supplementary Fig. 1. Cognitive and spatial navigation performance among the four diagnostic groups. *, p < 0.05. Supplementary Fig. 2. Associations between volumes of the basal forebrain using a 4-mm smoothing kernel and right hippocampal subfields and clinical measures. Partial correlation analyses were adjusted for sex, age, years of education, and total intracranial volume. *, p < 0.05; **, p < 0.01; ***, p < 0.001. The black * indicates results that survived multiple comparisons after FDR correction. [file 13195_2023_1233_MOESM1_ESM.zip › Supplementary_Figure_1.tif]

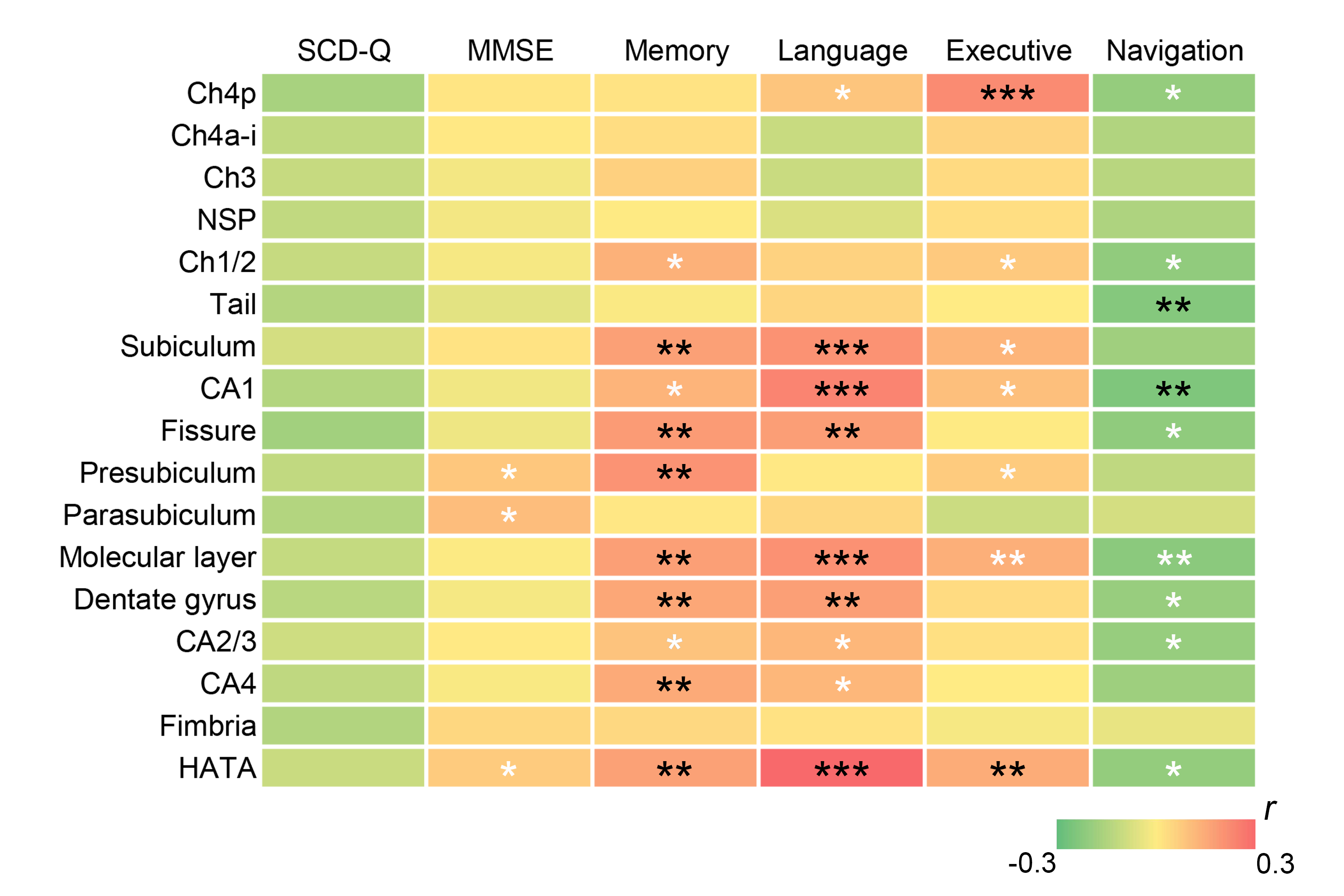

Supplement: Supplementary file 1 — Additional file 1: Supplementary Fig. 1. Cognitive and spatial navigation performance among the four diagnostic groups. *, p < 0.05. Supplementary Fig. 2. Associations between volumes of the basal forebrain using a 4-mm smoothing kernel and right hippocampal subfields and clinical measures. Partial correlation analyses were adjusted for sex, age, years of education, and total intracranial volume. *, p < 0.05; **, p < 0.01; ***, p < 0.001. The black * indicates results that survived multiple comparisons after FDR correction. [file 13195_2023_1233_MOESM1_ESM.zip › Supplementary_Figure_2.tif]
