# Supplementary material for: Spatial navigation is associated with subcortical alterations and progression risk in subjective cognitive decline
Source: Alzheimers Res Ther. 2023 Apr 25;15:86. doi: 10.1186/s13195-023-01233-6 (PMC10127414; doi:10.1186/s13195-023-01233-6)
Supplement: Supplementary file 2 — Additional file 2: Supplementary Table 1. Basal forebrain subfield volumes with a 4-mm FWHM smoothing kernel. [file 13195_2023_1233_MOESM2_ESM.docx]

**Supplementary Table 1 Basal forebrain subfield volumes with a 4-mm FWHM smoothing kernel**

|  | NC  (n = 77) | G-SCD  (n = 40) | B-SCD  (n = 40) | MCI  (n = 23) | *F* | *P* |
| --- | --- | --- | --- | --- | --- | --- |
| Ch4p | 97.47±9.53 | 98.04±11.48 | 92.60±7.43^bd^ | 90.51±6.48^ce^ | 4.904 | 0.003* |
| Ch4a-i | 150.91±10.74 | 153.16±14.32 | 145.84±13.56^d^ | 142.21±11.28^ce^ | 4.200 | 0.007* |
| Ch3 | 148.24±11.50 | 150.40±15.19 | 143.67±14.43 | 138.66±12.10^ce^ | 3.877 | 0.010* |
| NSP | 117.22±9.56 | 117.94±12.21 | 113.78±11.52 | 109.58±7.89^ce^ | 3.282 | 0.022* |
| Ch1/2 | 75.94±7.96 | 76.69±9.37 | 72.26±7.31 | 69.09±8.11^ce^ | 4.951 | 0.003* |

Data were presented as means±standard deviation. *, *p* < 0.05, FDR corrected, controlling for sex, age, years of education, and total intracranial volume. ^a~f^, post hoc analyses showed a significant difference between groups. ^a^: NC vs G-SCD; ^b^: NC vs B-SCD; ^c^: NC vs MCI; ^d^: G-SCD vs B-SCD; ^e^: G-SCD vs MCI; ^f^: B-SCD vs MCI.
