# Supplementary material for: Spatial navigation is associated with subcortical alterations and progression risk in subjective cognitive decline
Source: Alzheimers Res Ther. 2023 Apr 25;15:86. doi: 10.1186/s13195-023-01233-6 (PMC10127414; doi:10.1186/s13195-023-01233-6)
Supplement: Supplementary file 3 — Additional file 3: Supplementary Table 2. Basal forebrain subfield volumes based on different grouping methods of SCD. [file 13195_2023_1233_MOESM3_ESM.docx]

**Supplementary Table 2 Basal forebrain subfield volumes based on different grouping methods of SCD**

|  | Group by navigation ability | |  | Group by memory function | |  | Group by language function | |  | Group by executive function | |
| --- | --- | --- | --- | --- | --- | --- | --- | --- | --- | --- | --- |
|  | G-SCD  (n = 40) | B-SCD  (n = 40) |  | G-SCD  (n = 40) | B-SCD  (n = 40) |  | G-SCD  (n = 40) | B-SCD  (n = 40) |  | G-SCD  (n = 40) | B-SCD  (n = 40) |
| Ch4p | 86.40±9.27 | 81.96±6.34* |  | 84.85±8.85 | 83.51±7.55 |  | 84.95±8.71 | 83.41±7.70 |  | 85.79±7.48 | 82.57±8.66 |
| Ch4a-i | 144.01±13.01 | 137.13±11.32* |  | 141.37±13.39 | 139.78±11.88 |  | 140.15±13.55 | 141.00±11.74 |  | 142.86±11.19 | 138.29±13.64 |
| Ch3 | 136.77±13.27 | 130.98±11.29 |  | 134.70±13.20 | 133.04±12.05 |  | 133.21±13.42 | 134.54±11.82 |  | 136.04±10.85 | 131.71±13.90 |
| NSP | 104.93±10.01 | 101.44±9.79 |  | 103.48±10.34 | 102.89±9.75 |  | 102.66±10.07 | 103.71±10.01 |  | 104.03±9.00 | 102.33±10.94 |
| Ch1/2 | 67.36±8.00 | 63.56±6.17 |  | 66.56±7.85 | 64.37±6.74 |  | 65.81±8.49 | 65.11±6.10 |  | 66.47±6.61 | 64.45±7.98 |

Data were presented as means±standard deviation. *, *p* < 0.05, controlling for sex, age, years of education, and total intracranial volume.
