# Supplementary material for: Spatial navigation is associated with subcortical alterations and progression risk in subjective cognitive decline
Source: Alzheimers Res Ther. 2023 Apr 25;15:86. doi: 10.1186/s13195-023-01233-6 (PMC10127414; doi:10.1186/s13195-023-01233-6)
Supplement: Supplementary file 4 — Additional file 4: Supplementary Table 3. Follow-up data grouped by memory function. [file 13195_2023_1233_MOESM4_ESM.docx]

**Supplementary Table 3 Follow-up data grouped by memory function**

| Group by memory function | G-SCD  (n = 16) | B-SCD  (n = 23) | *t* | *P* |
| --- | --- | --- | --- | --- |
| Age | 64.31±5.17 | 66.04±6.06 | -0.930 | 0.358 |
| Sex (Male/Female) | 2/14 | 6/17 |  | 0.432 |
| Education | 12.31±2.82 | 12.28±2.55 | 0.034 | 0.973 |
| Outcome (converters/nonconverters) | 0/16 | 4/19 |  | 0.130 |
| Interval (days) | 479.38±128.07 | 577.96±176.02 | -1.912 | 0.064 |

Data were presented as means±standard deviation or number. The *p* values for sex and outcome were derived from Fisher's exact test, and statistics for other variables were derived from two sample *t*-test.
