# Supplementary material for: Spatial navigation is associated with subcortical alterations and progression risk in subjective cognitive decline
Source: Alzheimers Res Ther. 2023 Apr 25;15:86. doi: 10.1186/s13195-023-01233-6 (PMC10127414; doi:10.1186/s13195-023-01233-6)
Supplement: Supplementary file 5 — Additional file 5: Supplementary Table 4. Follow-up data grouped by language function. [file 13195_2023_1233_MOESM5_ESM.docx]

**Supplementary Table 4 Follow-up data grouped by language function**

| Group by language function | G-SCD  (n = 19) | B-SCD  (n = 20) | *t* | *P* |
| --- | --- | --- | --- | --- |
| Age | 64.68±6.37 | 65.95±5.08 | -0.688 | 0.496 |
| Sex (Male/Female) | 5/14 | 3/17 |  | 0.451 |
| Education | 13.05±2.20 | 11.58±2.85 | 1.805 | 0.079 |
| Outcome (converters/nonconverters) | 0/19 | 4/16 |  | 0.106 |
| Interval (days) | 536.47±151.30 | 538.50±178.78 | -0.038 | 0.970 |

Data were presented as means±standard deviation or number. The *p* values for sex and outcome were derived from Fisher's exact test, and statistics for other variables were derived from two sample *t*-test.
