# Supplementary material for: Spatial navigation is associated with subcortical alterations and progression risk in subjective cognitive decline
Source: Alzheimers Res Ther. 2023 Apr 25;15:86. doi: 10.1186/s13195-023-01233-6 (PMC10127414; doi:10.1186/s13195-023-01233-6)
Supplement: Supplementary file 6 — Additional file 6: Supplementary Table 5. Follow-up data grouped by executive function. [file 13195_2023_1233_MOESM6_ESM.docx]

**Supplementary Table 5 Follow-up data grouped by executive function**

| Group by executive function | G-SCD  (n = 21) | B-SCD  (n = 18) | *t* | *P* |
| --- | --- | --- | --- | --- |
| Age | 64.19±5.77 | 66.67±5.49 | -1.367 | 0.180 |
| Sex (Male/Female) | 5/16 | 3/15 |  | 0.702 |
| Education | 12.91±2.55 | 11.58±2.61 | 1.596 | 0.119 |
| Outcome (converters/nonconverters) | 1/20 | 3/15 |  | 0.318 |
| Interval (days) | 525.19±158.10 | 551.89±173.64 | -0.502 | 0.618 |

Data were presented as means±standard deviation or number. The *p* values for sex and outcome were derived from Fisher's exact test, and statistics for other variables were derived from two sample *t*-test.
